# Diabetes mellitus and associated risk factors among HIV infected patients on HAART

**DOI:** 10.1186/s12889-024-18265-8

**Published:** 2024-03-19

**Authors:** Lucy Kanyara, Rency Lel, Sheila Kageha, Joyceline Kinyua, Sophie Matu, Asiko Ongaya, Mary Matilu, Paul Mwangi, Vincent Okoth, Joseph Mwangi, Dama Olungae

**Affiliations:** Kenya Medical Research Institute, Nairobi, Kenya

**Keywords:** Diabetes mellitus, HIV, Antiretroviral therapy

## Abstract

Understanding the impact of disease associations is becoming a priority in Kenya and other countries bearing the load of infectious diseases. With the increased incidences of non-communicable diseases and the endemicity of infectious diseases in Sub-Saharan Africa, their co-existence poses significant challenges to patients, health workers and an overwhelmed health sector. Classical risk factors for diabetes such as physical inactivity and unhealthy diet may not solely explain the current trends, suggesting the role of novel risk factors including infections/inflammation. HIV and its treatment have been identified as potential contributors especially to patients with family history of confirmed diabetes cases. Co-infections frequently observed during HIV infection also significantly influence both the epidemiological and pathophysiological of the link between HIV and diabetes. Understanding the correlates of HIV and diabetes is crucial to inform management and prevention strategies of the twin infections. We therefore aimed to determine the prevalence of diabetes mellitus and risk factors in a population of HIV infected patients on HAART. This study determined the association of diabetes/impaired glucose regulation in the context of HIV-1. A cross-sectional study was conducted at a comprehensive care clinic in Nairobi (Kenya). Participants were screened for diabetes and impaired glucose regulation using random blood glucose and glycated haemoglobin (HbA1c) This paper describes the prevalence of diabetes mellitus in Human Immunodeficiency Virus positive individuals and the associated risk factors. We have demonstrated that family history is a risk factor for diabetes. While age and BMI are known risk factors, they were not associated with diabetes in this study.

**Supplementary Information:**

The online version contains supplementary material available at 10.1186/s12889-024-18265-8.

## Introduction

Around 37.7 million individuals worldwide were living with HIV while 27.5 million people were accessing antiretroviral therapy at the end 2020 (UNAIDS global HIV and AIDS statistics [1]. In Kenya, around 1,500,000 persons were infected with HIV, with an adult prevalence rate of roughly 4.9% and 93% of those infected on treatment target of 90.90.90 initiative [KENPHIA. 2019]. In today’s world, HIV and noncommunicable diseases (NCDs) have become major public health concerns globally. HIV/AIDS and NCDs have been linked in studies, which could be due to direct effects of HIV or indirect effects of medication regimens [2]. Diabetes has been linked to the use of HAART, as well as HIV in general, among HIV patients (PLWHIV). With HAART and better HIV care, HIV-infected individuals are living longer, hence an increase in NCDs is unavoidable. Non–AIDS–defining diseases have increased in the era of highly active antiretroviral therapy. HIV-infected people on HAART have a greater prevalence of diabetes mellitus and develop it at a younger age than HIV-negative people [2, 3].

In today’s world, HIV and noncommunicable diseases (NCDs) have become major public health concerns globally. HIV/AIDS and NCDs have been linked in studies, which could be due to direct effects of HIV or indirect effects of medication regimens [2]. Diabetes has been linked to the use of HAART, as well as HIV in general, among HIV patients (PLWHIV). With HAART and better HIV care, HIV-infected individuals are living longer, hence an increase in NCDs is unavoidable. Non–AIDS–defining diseases have increased in the era of highly active antiretroviral therapy. HIV-infected people on HAART have a greater prevalence of diabetes mellitus and develop it at a younger age than HIV-negative people [2, 3].

Diabetes Mellitus (DM) is an emerging global health problem taking root within the continent in the last decade. Changing lifestyles and dietary habits has caused an upward trajectory of DM and other NCDs which were historically not considered an African problem. The global burden of NCDs is impactful causing two thirds of global deaths [4] and in 2017, global incidence, prevalence, death, and disability-adjusted life-years (DALYs) associated with diabetes were 22.9 million, 476.0 million, 1.37 million, and 67.9 million, with a projection to 26.6 million, 570.9 million, 1.59 million, and 79.3 million in 2025, respectively [5, 6].

The rise of non-communicable diseases (NCD) such as diabetes in conjunction with infectious diseases intensifies pressure on already weak health systems. Vertical approaches can no longer be used in national healthcare practices. There needs to be multidirectional approaches that address the dual burden of infectious and non-communicable diseases.

Studies have shown conflicting outcomes on metabolic syndrome and glucose dysregulation among HIV-infected individuals, but most suggest an association between HIV infection and diabetes mellitus. Antiretroviral therapy for HIV may increase the risk of metabolic syndrome (the clustering of abdominal obesity, hyperglycemia, dyslipidemia and hypertension) [7] and thus predispose to communicable diseases. Some of the unexpected consequences for people on HAART, due to defects of this lipid metabolism, include an increased frequency of glucose intolerance or DM [8, 9]. Research done in high-income countries suggest that, although antiretroviral therapy (ART) can suppress viral load, the health of HIV-infected people is not completely restored, and people living with HIV have higher risk of diabetes than HIV-uninfected.

Identifying co-morbidities and their overlapping management poses a significant challenge for health programming especially in Sub-Saharan Africa where data on dual and triple infections and their synergistic roles as well as resource needs is limited. This paper proposes to assess the prevalence of diabetes mellitus and antiretroviral therapy use and other associated risk factors among HIV positive adults in Kenya.

## Methods

### Study setting, participants and design

The study was conducted at the Comprehensive Care Clinic (CCC) in KEMRI CRDR within Nairobi County. Sample collection occurred between May 2020 to August 2020 where 325 HIV infected individuals were enrolled. Random sampling was used to select the study participants. Socio-demographic and medical data and information on risk factors was collected from eligible consenting participants using a modified WHO STEPS questionnaire to capture data.

### Inclusion and exclusion criteria

HIV infected patients aged above 18 years that consented to participate. We further excluded patients that were pregnant, and those that refused to consent to participate for personal reasons.

### Sample collection

2 ML peripheral venous blood was drawn from each study participant using an EDTA vacutainer tube for Random blood and HB1Ac. Blood was transported to the laboratory for subsequent separation, analysis and storage.

### Demographic data Collection

Using structured questionnaires, demographics information and behavioral risk factors information was collected. These included data on alcohol consumption, diet practice of the patients, physical activity status, history of hypertension and diabetes in the family. Information on ART usage and adherence was confirmed from patients’ cards and clinical data. All the data was captured using a study survey tool on ODK.

### Anthropometry and body composition

At the clinic, anthropometric measurements were taken. These included measuring the weight to the nearest 0.1 Kg. The height was also measured and body mass Index (BMI) calculated as weight (kg)/height (m)2 and WHO cut-off values were used to classify participants as underweight (BMI < 18.5 kg/m^2^), normal weight (BMI 18.5–24.99 kg/m^2^), and overweight (BMI 25–30 kg/m^2^) and obese (> 30 kg/m^2^). Waist circumferences were taken and ≥ 94 cm in males and ≥ 80 cm in females considered elevated.

### Analytical methods

Random capillary Blood Sugar (RBS) was determined at the CCC on recruitment for all study participants to identify persons with elevated or reduced sugar levels, an indicator of glucose metabolism. This was achieved using a glucometer according to the manufacturer’s instructions. If RBS was above 7.8.mmol/l the blood was further subjected to Glycated Hemoglobin (HbA1c) screening using GREENCARE A1c One Step HbA1c measuring system Model. RT-100(Green Cross Medical Science Corp, South Korea) point of care machine. Normal levels were interpreted to be within the range Hb1Ac Prediabetes (6.0–6.4%) Hb1Ac had a chance of getting Diabetes while 6.5% or higher were reported as positive for Diabetes.

### Data Analysis

Descriptive statistics were used for the demographic and clinical data. Retrospective data of HIV viral load was accessed for the patient files from the Comprehensive care clinic. The results obtained in this study were analyzed statistically using ANOVA and regression with RStudio.

### Ethical approval

Ethical approval was obtained from the Scientific Ethical Research Unit (SERU) of the Kenya Medical Research Institute (KEMRI). Additional approval for sample collection was obtained from the Center for Respiratory Disease Research (CRDR), the site of sample collection. The study only involved the patients who agreed to participate and provided written informed consent.

## Results

This study determined the association of diabetes/impaired glucose regulation in the context of HIV-1. A cross-sectional study was conducted at a comprehensive care clinic in Nairobi (Kenya). Majority of the study subjects were male (66%) with more than half being married. About 73.9% had secondary/tertiary education level while most of the participants had formal employment. Participants were screened for diabetes and impaired glucose regulation using random blood glucose and glycated hemoglobin (HbA1c). 326 participants were enrolled in the study and patients’ history of diabetes against current Diabetes diagnosis (*N* = 306). From the Pearson’s square test (Pr < 0.05), there is a strong correlation of HIV patients testing positive for Diabetes having a family member being positive previously (Pr = 0.009) There was a correlation of family history of diabetes with testing positive for diabetes as driven by HbA1c test. The data showed that there is a relationship between blood sugar and viral load P Value of 0.0422971. Table 1 presents the demographic data of study participants 40% of the participants were above 50 years with the majority of them being men. 57.5% were married with 73.9% with Secondary or tertiary education.

**Table 1:** Socio-demographic and lifestyle characteristics N = 306

Variable	Category	Frequency (%)
Age (Years)	18–30	32 (10.5)
	31–40	47 (15.4)
	41–50	103 (33.7)
	> 50	124 (40.5)
Sex	Male	202 (66.0)
	Female	104 (34.0)
Marital status	Married	176 (57.5)
	Widowed	27 (8.8)
	Co-habiting	1 (0.3)
	Single/Not married	102 (33.3)
Education Status	No education	4 (1.3)
	Primary	75 (24.5)
	Secondary/Tertiary	226 (73.9)
Occupation	Not employed	42 (13.7)
	Informal	69 (22.5)
	Formal/skilled	91 (29.7)
	Retired	10 (3.3)
	Others	94 (30.7)

Table 2 Above shows the present’s diagnosis of diabetes which was grouped in the age bracket of the study participants. Random blood sugar was carried out in all participants, the majority were > 50 years. 25% presented with hypoglycemia with lowest being 1.0mmo/l while only 11 participants with hyperglycemia with blood sugar of 20.0mmol/l. The random blood sugar was confirmed usingHbA1c with prevalence of diabetes 3.5% in the study participants.

**Table 2:** Diagnosis of diabetes against different age brackets of patients under HIV care

Age (Years)	< 3.5 mmol/l Hypoglycemia	3.5-7.8mmol/l Normal	> 7.8mmol/l Hyperglycemia	Diabetes ( > = 6.5)
18–30	12	18	1	0
31–40	16	28	0	0
41–50	22	72	3	4
> 50	27	84	7	11

**Fig. 1:**
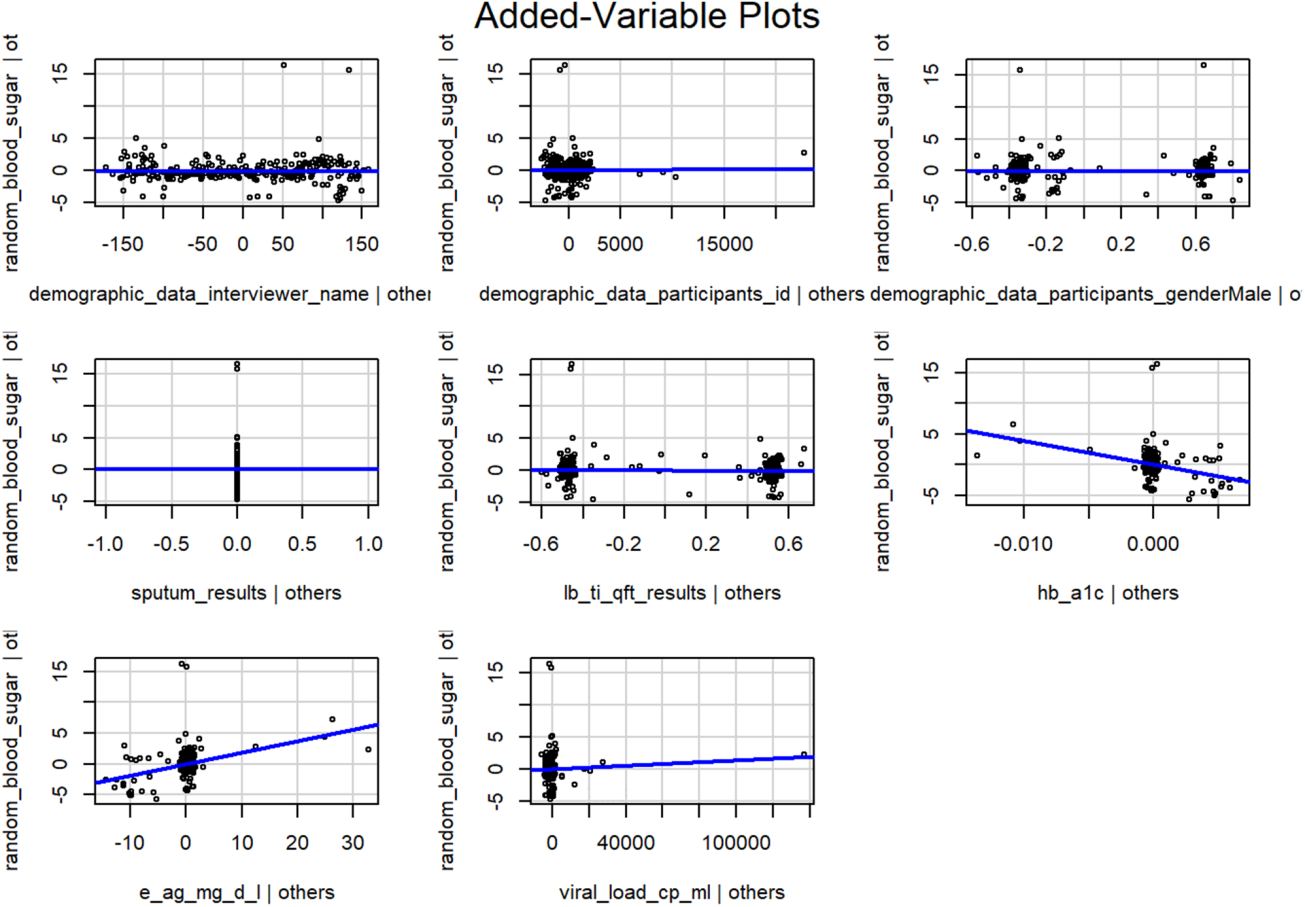
Prevalence of Viral Load by Gender

Fig. 1 is a scatter plot is confirmed by the gender bar graph above. There was a positive correlation between viral load and random blood sugar in males but there is a negative correlation for the same in females.

**Fig. 2:**
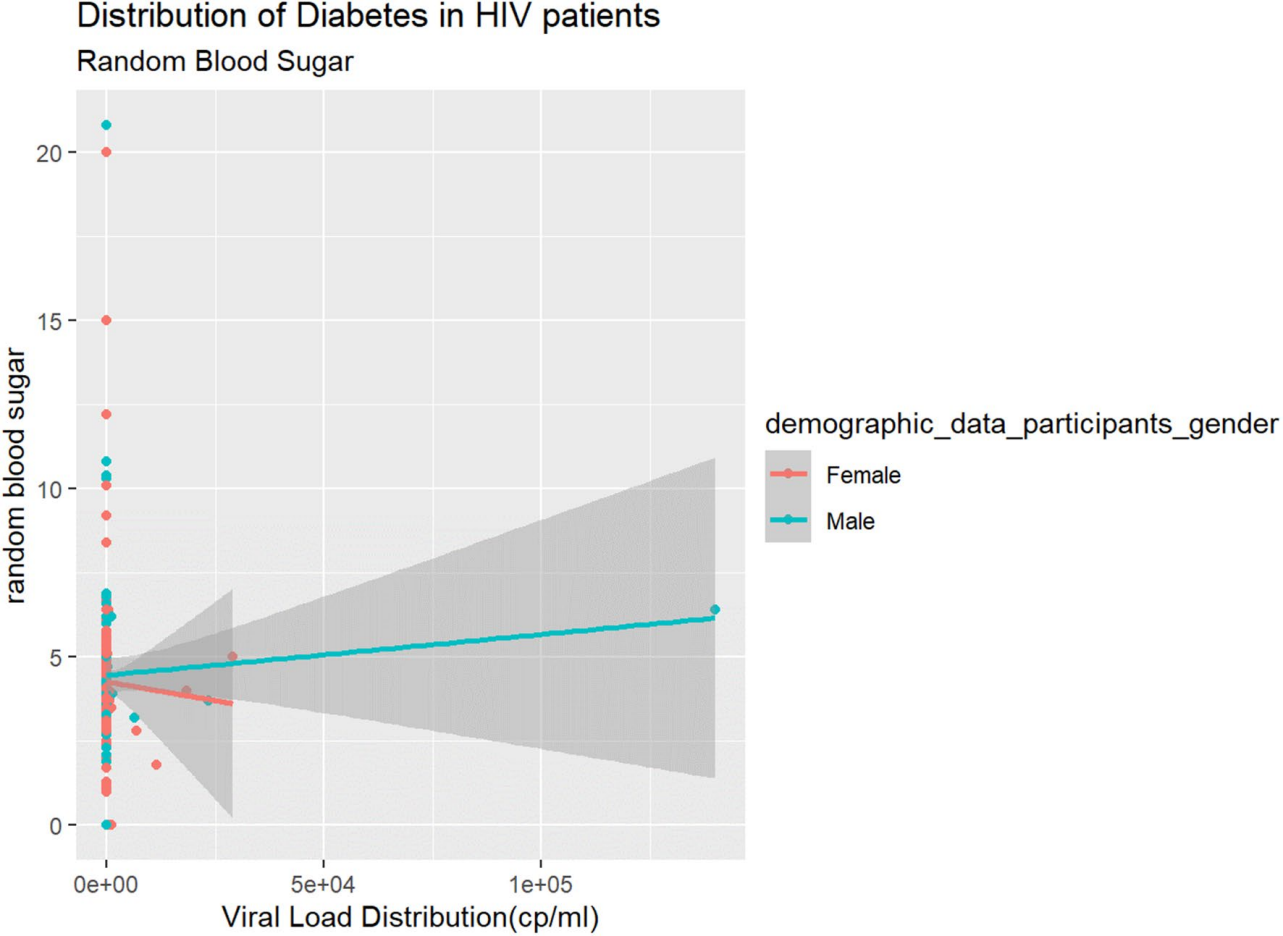
Distribution of random blood sugar

Fig. 2: The data demonstrated a positive correlation between viral load and random blood sugar in males but surprisingly there is a negative correlation for the same in females. 

## Discussion

The study was conducted to determine the prevalence and correlates of diabetes in HIV patients where 306 HIV-1 patients attending comprehensive care clinic in Nairobi. The results indicated that 25% were hypoglycemic and 11 were hyperglycemic and a further HbA1C was done to confirm the test showing 3.5% prevalence of diabetes. Random blood sugar was carried out in all participants, the majority were > 50 years. 25% presented with hypoglycemia with lowest being 1.0mmo/l while only 11 participants with hyperglycemia with blood sugar of 20.0mmol/l. The random blood sugar was confirmed using HbA1c with prevalence of diabetes being 3.5% in the participants(Fig. 2). The data demonstrated a positive correlation between viral load and random blood sugar in males but surprisingly there is a negative correlation for the same in females. The data suggested that Men High blood VL (Fig. 1) is inversely correlated with high sugar Women although the link between viral load and random blood sugar in males was positive, it was negative in females due to the high blood VL and normal sugar levels

### Study limitations

The study has limitations and primarily, it is a cross-sectional study and has had no long-term follow-up to conclusively state the associations between diabetes mellitus and HIV. The inclusion of PLHIV visiting a specific clinic may have not been representative of the larger and general population, nonetheless the results were reflective of communities with similar characteristics. The small number of participants may have limited associations due to the limited statistical power.

## Conclusion

Despite the growing prevalence of chronic illnesses like diabetes in Sub-Saharan African countries, there is minimal screening of HIV infected individuals for these conditions. Non-communicable diseases should be incorporated within management of HIV patients.

Further research is key in determining the disease burden in HIV positive patients and for integrating HIV and NCDs care.

### Electronic supplementary material

Below is the link to the electronic supplementary material.

Supplementary Material 1: Raw data

## Data Availability

The article and its supplemental files contain the information needed to support its findings. The lead author Lucy Kanyara will provide more information upon reasonable request.
